# Effect of Choline Supplementation on Rapid Weight Loss and Biochemical Variables Among Female Taekwondo and Judo Athletes

**DOI:** 10.2478/hukin-2014-0009

**Published:** 2014-04-09

**Authors:** Gehan Elsawy, Osama Abdelrahman, Amr Hamza

**Affiliations:** 1 Faculty of Physical Education, Zagazig University, Egypt.; 2 Faculty of Physical Education, Mansoura University, Egypt.; 3 Faculty of Medicine, Sports Unit, Zagazig University, Egypt.

**Keywords:** Choline supplementation, body mass reduction, biochemical variables, female athletes, combat sports

## Abstract

Taekwondo and judo competitions are divided into weight categories. Many athletes reduce their body mass a few days before competition in order to obtain a competitive advantage over lighter opponents. To achieve fast body mass reduction, athletes use a number of nutritional strategies, including choline supplementation. The goal of this study was to identify the effects of choline supplementation on body mass reduction and leptin levels among female taekwondo and judo athletes. Twenty-two female athletes (15 taekwondo and 7 judo athletes) were selected from different weight categories and divided into two groups, according to weight. The players in the experimental group took choline tablets for one week before a competition. The results revealed significant differences between pre- and post-competition measurements of leptin, free plasma choline, urine choline and urine malondialdehyde levels; body mass was also reduced in the post-competition measurements. In conclusion, choline supplementation could rapidly reduce body mass without any side effects on biochemical levels or static strength.

## Introduction

Athletes are continuously looking for ways to raise the level of their performances and exceed their individual capacities in order to achieve sporting success. Since increases in training loads cannot meet the aspirations of athletes, competitors search for methods to improve motor abilities with minimal side effects.

Athletes use dietary supplements (DS) in order to increase energy, maintain strength, health and immune system function, enhance performance, and prevent nutritional deficiencies (Braun et al., 2009; Tian et al., 2009). An increase in DS use has been observed in a variety of sports, especially among elite athletes (Berglund, 2001; Huang et al., 2006). Many athletes have turned to various dietary strategies, including the use of various DS or sports supplements, which they presume to be effective, safe and legal (Melvin, 2004). Several studies estimate that supplement use among athletes is common, with a prevalence ranging from 59 to 88%. Multivitamins, minerals, proteins and energy drinks are the most common products consumed (Braun et al., 2009; Tian et al., 2009).

Some athletes strive to maintain a continuously reduced body mass due to the requirement of the sporting modality. For example, athletes who struggle to compete in lighter weight categories against smaller and weaker opponents reduce their body mass days before competition (Brownell and Steen, 1987).

Steen and Brownell (1990) reported that, among American college athletes fighting in the Olympics, 41% lose 5 to 9.1 kg each week during the competitive season. Some athletes reported losing 22.7 kg, and others reported reducing their body mass 60 times in one season. To achieve the necessary body mass reduction, these athletes use a series of procedures that may affect both health and athletic performance (Brownell and Steen, 1987; American College of Sports Medicine, 1996; Zambraski et al., 1975). The most common strategies to reduce body mass are severe food restriction, conducting strenuous exercise, and dehydration. Dehydration is often achieved by restricting fluid intake, using saunas, and training with or without warm clothes (Oppliger et al., 2003). According to Guilherme et al. (2007) the most frequently used methods of losing weight rapidly were dehydration, decreased energy intake and decreased consumption of sweets and fats.

Further, some athletes report inducing vomiting and taking laxatives and diuretics in attempts to reach the weight of a competitive category (Steen and Brownell, 1990). To perform well in judo tournaments, athletes must possess a high level of technical and tactical abilities, as well as strength, aerobic capacity, flexibility, and anaerobic power (Little, 1991). In the top levels of competition, in which the technical-tactical abilities of athletes are generally equivalent, the importance of adequate physical preparation is more pronounced. Small changes in any variable that influences performance can determine the final outcome of a competition. Thus, the practice of rapid body mass can be counterproductive to performance, indicating that athletes may be competing with an impaired physical potential.

Individuals who are weight-reduced or leptin-deficient have a lower energy expenditure coupled with higher hunger and disinhibition and/or delayed satiation compared with never-weight-reduced control subjects. Exogenous leptin inhibits feeding in congenitally leptin-deficient humans and reduced leptin signaling may reduce the expression of feeding inhibition in humans (Kissileff et al., 2012).

Most weight-loss supplements contain caffeine, which increases the metabolic rate. This practice is not healthy, since caffeine also causes nervousness, insomnia, nausea and increased blood pressure.

The amine choline is a member of the vitamin B-complex family and it is naturally produced by the body to burn fat. Choline is also found naturally in a variety of foods and its Recommended Dietary Allowance (RDA) is grouped with the B vitamins (National Academy of Sciences, 2000). Choline is responsible for the production of the neurotransmitter acetylcholine, which is released in the brain and at neuromuscular junctions; acetylcholine is responsible for many physiologically important events, including providing structural integrity and signaling roles in cell membranes, and it is a major source of methyl groups via its metabolite and cholinergic neurotransmission (acetylcholine synthesis). Reduced acetylcholine in the nervous system has been theorized to contribute to the development of fatigue. Plasma choline levels are significantly reduced following marathon running, and choline supplementation has been hypothesized to prevent fatigue in athletes. Still, there are no definitive studies that justify choline supplementation (Kanter and Williams, 1995).

Research is needed to clarify the magnitude of rapid body mass reduction among Egyptian judokas, in order to identify the scientific basis and justification for such practices. Thus, this study aimed to identify the effects of choline supplementation on body mass reduction and leptin levels among female’s taekwondo and judo athletes.

## Material and Methods

### Participants

Twenty-two female athletes (15 taekwondo) and (7 judo) from different weight categories participated in this double-blind clinical trial, which was conducted in 2011. The athletes were divided into two groups, according to their body mass; the experimental group contained ten female athletes, and the control group twelve female athletes. At the time of enrollment, all the subjects were healthy, according to a medical information questionnaire, and none of the subjects had any specific dietary restrictions. Exclusion criteria included the use of any medication or supplement during the previous six months. For one week prior to a competition, the athletes in the experimental group took choline tablets (1.0 g) twice daily with a meal, equaling a total daily dose of 2.0 g. The control group received a placebo, and they participated in usual training (with 75% training intensity) at the same time as the choline group four times per week.

According to Anni et al. (2011), choline supplementation appears to be safe and the authors recommend taking approximately 2.5 g one hour before a prolonged exercise session. The effective dose in sport studies is 0.2 g phosphatidylcholine 90% per kg of the body mass, which equals 2.1 g of choline for an 80-kg athlete. There is no requirement for a loading or maintenance phase and choline supplementation up to one hour before exercise has been shown to be effective in reducing fatigue.

### Procedures

Collection of blood and urine samples

Subjects provided fasting blood samples before the intake of choline and after one week. A blood sample (3 ml) was collected in an EDTA-containing Vacutainer tube and centrifuged at 4°C at 3000×g for 10 minutes. The plasma was decanted and frozen at − 70°C prior to analysis. Subjects also collected their urine for the subsequent five-hour period. The urine was kept refrigerated during the collection period. Plasma was analyzed for leptin and free choline by mass spectrophotometry. Urine malondialdehyde (MDA) and urine choline were measured by a fluorometric assay and visually-read colorimetric assay.

### Measurement of body height, body mass and fat content

All anthropometric data, including age, body mass, body height, and body fat, were measured before collecting blood samples. Body height of the subjects was measured with a metal scale with 0.1 cm sensitivity, and body mass was measured with a digital scale with 0.1 kg sensitivity. Body fat percentages were measured with the Tanita Bioimpedance BC-418 (Tokyo, Japan).

### Assessment of static strength tests

A back dynamometer was used to measure static leg strength. The subjects stood on the dynamometer platform and crouched to the desired leg position. They were connected to the dynamometer with a waist-strap. When directed, the subjects exerted a maximum force upward by extending their legs. They kept their backs straight, heads erect and chests high. Three trials were allowed for each subject and the best score was recorded. Subjects were allowed to rest between each trial.

### Statistical Analysis

All statistical analyses were calculated by the SPSS statistical package. The results are reported as means and standard deviations (SD). Differences between two groups were reported as mean difference ±95% confidence intervals (mean diff ± 95% CI). The Student’s t-test for independent samples was used to determine the differences in physical and biochemical parameters between the two groups. The level of significance was set at p<0.05.

## Results

Table 1 shows the age and training experience of the subjects. No significant differences were observed in the age, anthropometric characteristics or training experience of the subjects in the two groups.

Table 2 shows significant differences that were observed in levels of leptin, free plasma choline, urine MDA and body fat for the choline group. No significant differences were observed in urine choline, BMI, or static strength between the two groups.

## Discussion

The goal of this study was to identify the effects of choline supplementation on body mass reduction and leptin levels among female taekwondo and judo athletes. The results indicated that choline supplementation reduced leptin levels (6.11 ± 0.76 to 5.78 ± 0.94, percent change 5.71%), body fat (%) (18.76± 2.71 to 16.84± 3.08, percent change 10.23%), and BMI (24.97 ± 3.3 to 21.93 ± 5.6, percent change 12.17%). These results support the hypothesis that choline could be used in conjunction with normal athletic training to lose weight rapidly in the week before a competition.

Body mass alone is not a good indicator of health for athletes; there are two important factors to be considered: body fat percentage and muscle mass. In general, reducing body fat is equivalent to losing weight. When an athlete eats more calories than he or she burns, the body fat stores will increase. If an athlete does the opposite (burns more than eats), his body fat will decrease.

Hanin et al. (1987) reported that choline plays an important role in the metabolism of fat; it breaks fat down for use as an energy source. This action of choline makes it valuable in preventing conditions like fatty liver or excess fat in the blood. Choline’s efficient metabolism of fats has also been linked to a greater level of satiety, which, in turn, leads to a decreased consumption of calories, resulting in overall weight loss. According to studies conducted by the Louisiana State University Pennington Biomedical Research Center (2007), the addition of choline-rich eggs to breakfast helped obese patients on a low-fat diet lose weight compared to patients who ate a bagel for breakfast with the same number of calories.

Several studies have shown that choline supplementation significantly increases plasma choline levels (Wurtman et al., 1977; Hirsch et al., 1978). Ingesting choline resulted in higher peak serum choline concentrations (265% above normal levels) and these levels remained elevated for 12 hours (Wurtman et al., 1977).

Another important result of our study is the significant reduction in the urine malondialdehyde (MDA) secretions after choline supplementation. These findings demonstrate the quality of choline as an antioxidant.

During exercise, the conversion of fat and sugar into energy occurs through a process known as oxidation. During this process, most of the oxygen combines with hydrogen to produce water. However, approximately 5% of the oxygen forms free radicals (Mark, 2012). Damage from free radicals can cause tissue damage and lead to certain cancers. Free radical damage has become more prevalent in our society, as evidenced by the increase in cancer deaths in recent years. Research shows that mental stress is one of the highest causes of free radicals.

Dekkers et al. (1996) concluded that dietary supplementation with antioxidant vitamins has favorable effects on lipid peroxidation and exercise-induced muscle damage. Evans (2000) noted that several antioxidant vitamins have been shown to decrease the exercise-induced increase in the rate of lipid peroxidation, which could help prevent muscle tissue damage.

## Practical Applications

Choline supplementation in female taekwondo and judo athletes can improve lipid metabolism and be used as an antioxidant, as well as encourage rapid body mass reduction.

## Figures and Tables

**Table 1:** Anthropometric characteristics of the studied athletes

**Variables**	N	Age (years)	Body mass (kg)	Body height (cm)	Body mass index (kg/m^2^)	Body fat (%)	Training experience (years)
**Choline group**	10	20.43±2.45	75.94±10.23	176.88±9.95	24.97 ± 3.3	18.76 ± 2.71	8.23±2.11
**Control group**	12	23.43±4.68	76.23±11.66	177.36±10.03	24.88 ± 3.1	18.52 ± 2.36	7.65±1.93
**Total**	22	21.15 ± 1.9	76.54 ± 9.1	175.22 ± 8.2	24.93 ± 3.3	18.64 ± 2.4	8.15 ± 3.4

**Table 2:** Biochemical variables and static strength for the control and experimental groups

**Variables**	**Control**			**Experimental**			***p***
	pre^*^	post^*^	**change%**	pre^*^	post^*^	**change%**	
Leptin (ng/ml)	6.02 ± 0.64	5.90 ± 0.53	1.99	6.11 ± 0.76	5.78 ± 0.94	5.71	0.05
Free plasma Choline Nmol /L	7.16 ± 2.57	7.17 ± 2.59	0.14	7.03 ± 2.11	8.23 ± 3.61	17.07	0.01
Urine Choline Nmol /L	8.58±2.89	8.43 ± 3.22	1.75	8.22 ±3.51	8.05 ± 3.73	2.07	ns
Urine MDA Mmol /L	12.14 ± 1.23	11.39 ± 1.35	6.18	12.67 ± 1.56	11.06 ± 1.98	12.62	0.01
Body fat (%)Body Mass	18.52± 2.36	17.77± 2.97	4.05	18.76± 2.71	16.84± 3.08	10.23	0.01
Index (kg/m^2^)	24.88 ± 3.1	22.91 ± 3.4	7.92	24.97 ± 3.3	21.93 ± 5.6	12.17	ns
Leg strength (kg)	92.38 ±5.67	94.36± 8.34	2.14	93.02 ±7.68	95.78± 9.46	2.97	ns
Back strength (kg)	75.16± 6.08	77.29± 6.17	2.83	77.35± 8.34	79.29± 9.07	2.51	ns

* All results are presented as mean ± SD
